# Utilizing an Ex Vivo Skin Penetration Analysis Model for Predicting Ocular Drug Penetration: A Feasibility Study with Curcumin Formulations

**DOI:** 10.3390/pharmaceutics16101302

**Published:** 2024-10-06

**Authors:** Christian Raab, Stefan Brugger, Jara-Sophie Lechner, Geisa Nascimento Barbalho, Taís Gratieri, Priyanka Agarwal, Ilva D. Rupenthal, Cornelia M. Keck

**Affiliations:** 1 Department of Pharmaceutics and Biopharmaceutics, Philipps-Universität Marburg, Robert-Koch-Str. 4, 35037 Marburg, Germany; christian.raab@pharmazie.uni-marburg.de (C.R.);; 2 Buchanan Ocular Therapeutics Unit, Department of Ophthalmology, Aotearoa-New Zealand National Eye Centre, Faculty of Medical and Health Sciences, The University of Auckland, Auckland 1142, New Zealandgeisabarbalho@gmail.com (G.N.B.); p.agarwal@auckland.ac.nz (P.A.); 3 Laboratory of Food, Drugs and Cosmetics (LTMAC), University of Brasilia, Brasilia 70910-900, Brazil; tgratieri@gmail.com

**Keywords:** curcumin, dermal penetration, corneal penetration, bioavailability, image analysis, ex vivo models

## Abstract

**Objective:** This study aimed to investigate the feasibility of using the digital image processing technique, developed to semi-quantitatively study dermal penetration, to study corneal penetration in an ex vivo porcine eye model. Here, we investigated various formulation strategies intended to enhance dermal and corneal bioavailability of the model hydrophobic drug, curcumin. Methods: Several formulation principles were explored, including oily solutions, oily suspensions, aqueous nanosuspension, micelles, liposomes and cyclodextrins. The dermal penetration efficacy was tested using an ex vivo porcine ear model previously developed at Philipps-Universität Marburg with subsequent digital image processing. This image analysis method was further applied to study corneal penetration using an ex vivo porcine whole-eye model. Results: For dermal penetration, oily solutions, oily suspensions and nanosuspensions exhibited the least penetration, whereas liposomes and cyclodextrins showed enhanced penetration. Corneal curcumin penetration correlated with dermal penetration, with curcumin loaded into cyclodextrins penetrating the deepest. Conclusions: Overall, our study suggests that the image analysis method previously developed for ex vivo skin penetration can easily be repurposed to study corneal penetration of hydrophobic drugs.

## 1. Introduction

Drug delivery aims at transporting active pharmaceutical ingredients (API) at the correct dose, at the appropriate time, to the intended site of action. To realize this objective, a variety of different formulation principles containing various excipients, which aid in the formulation of APIs, are available today. However, despite the effective formulation of APIs, it is also crucial to have powerful test methods that can accurately predict the in vivo efficacy of a formulation. These models should be able to reliably distinguish between high-performing and low-performing formulations, ideally at a preliminary stage of development.

The burden of ocular diseases is rapidly increasing, with the World Health Organization estimating that over 314 million people have visual impairment globally [1]. The global market for ophthalmic drugs has been estimated to be a staggering 29.2 million dollars, with over 90% of ophthalmic formulations being eyedrops targeting anterior segment conditions [2,3]. In the area of ocular drug delivery, only limited models are available to predict the bioavailability of ophthalmic formulations in a time- and cost-effective manner [4,5]. The unique anatomical features of the eye with its multiple barriers and complicated fluid dynamics render this organ difficult to simulate. Today’s available test models include in vitro methods, e.g., release studies utilizing Franz diffusion cells or dialysis tests, 2D and 3D cell culture models and ex vivo models that use excised animal corneas as a membrane [4,6]. New developments also use whole eyeballs and analyze the penetrated amount of the API after extraction from the ocular tissue [4,7]. However, drug extraction is often time-consuming, and requires experienced personnel and high-end analytical equipment for the quantification of the API. Drug extraction and analysis by high-performance liquid chromatography or liquid chromatography–mass spectrometry is typically employed for drug quantification in ocular tissues [8]; however, these techniques only provide quantitative bioavailability data without giving any insight into drug penetration depth and distribution within the tissue. Fluorescent and/or confocal microscopy has also been used in the past to visualize drug distribution in ocular tissues [9,10].

**Figure 1:**
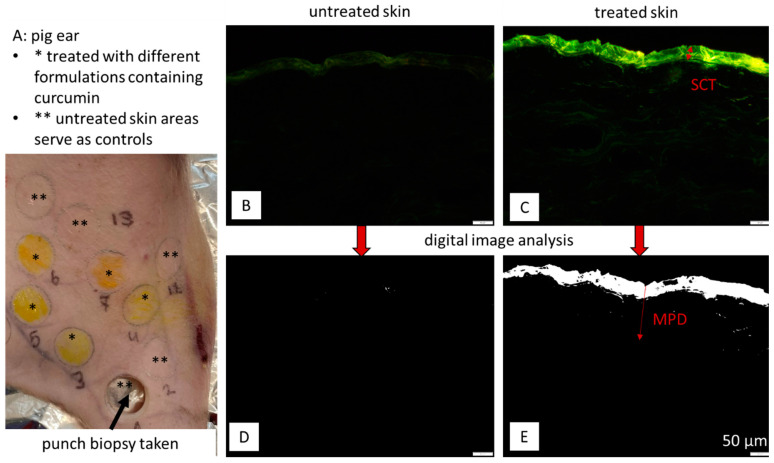
Scheme of dermal penetration testing with the ‘Marburg skin penetration’ model. (**A**) Fresh pig ear with areas for treatment. (**B**,**C**) Cross-sectional images of skin biopsies taken by inverted epifluorescence microscopy. (**D**,**E**) Images after digital image processing, where an automated threshold was applied to subtract the autofluorescence of the skin. The remaining pixels represent the penetrated API. SCT—stratum corneum thickness; MPD = mean penetration depth. Scale bar = 50 µm.

A similar situation was found for the testing of dermal formulations until a simple and cost-effective ex vivo method that forecasts the dermal penetration efficacy of an API in skin semi-quantitatively was developed in 2021 [11]. The ‘Marburg skin penetration model’ uses freshly slaughtered pig ears on which the formulations are applied (Figure 1A). After the application of the test formulations, the skin is treated to simulate real-world situations. In most cases, this includes the application of the formulation in a finite dose setup, as well as incubation of the ears at 32 °C for different periods of time. More complex studies include other skin treatments, e.g., massage, UV radiation and microneedle treatments. After the skin treatment and the intended incubation time, punch biopsies of the treated skin sections are taken (Figure 1A), which are then cryo-sectionized into 20 µm thick vertical skin cuts from which images are taken using an inverted epifluorescence microscope (Figure 1B,C).

From the pictures, the stratum corneum thickness (SCT), as a sensitive measure of the skin hydration status, can be measured (Figure 1C). In the next step, the images obtained are subjected to digital image analysis to subtract the autofluorescence of the skin from the original image. After the subtraction of the skin autofluorescence, the remaining bright pixels in the image visualize and represent the penetrated API in the skin (Figure 1D,E). Digital image analysis is then used to measure the number of the remaining pixels in each image. The number of pixels in each image is expressed as the mean grey value per pixel [MGV/px]. Untreated skin has a low MGV/px (Figure 1D), whereas skin areas that were treated with the API show higher MGV/px (Figure 1E). Consequently, images with high MGV/px are images from formulations with good dermal penetration of the API, whereas pictures with lower MGV/px are derived from skin that was treated with formulations with lesser dermal penetration efficacy for the API. In addition to the number of pixels, the mean penetration depth (MPD) of the API can be assessed by measuring the distance between the skin surface and the most distant pixels from the skin surface left after digital image processing (Figure 1E).

The method is semi-quantitative and allows for a fast, cost-effective and easy-to-perform bioavailability testing of dermal formulations [11]. Moreover, in contrast to typically used analytical techniques, like HPLC and LCMS, it enables us to visualize the distribution of active compounds within the tissue, which helps to better understand, for example, drug distribution patterns or the drug’s affinity for different layers of the tissue. In addition, it visualizes the tissue morphology and any change in tissue structure in response to formulations, excipients and/or drugs.

While distinct differences are present between corneal and dermal anatomy and tissue morphology [12,13], it has often been shown that several drug penetration principles can be extended from the skin to the cornea and vice versa [14,15], due to similar tissue characteristics, osmotic gradients and regeneration capacity [16,17,18]. As such, similar formulation strategies can often be employed to enhance corneal and dermal penetration [19,20,21]. Furthermore, it was hypothesized that the semi-quantitative digital image analysis method used to study skin penetration could also be applied to evaluate drug penetration in corneal tissue. These hypotheses were tested in three parts in the present study.

In the first step, different ophthalmic formulations, containing curcumin, a poorly soluble drug, as a model drug [22], were produced and characterized regarding their physico-chemical properties. Curcumin is a cost-effective and inherently fluorescent bioactive known for its anti-inflammatory, antibacterial and antiproliferative effects and properties [23,24,25]. However, its hydrophobic nature renders its formulation and delivery rather difficult. The formulations selected to enhance the bioavailability of curcumin were two classical formulation principles, i.e., an oily solution and an oily suspension of curcumin [9], and four innovative formulations, including an aqueous nanosuspension (nanocrystals), curcumin entrapped in micelles, liposomes and cyclodextrins. All formulations contained excipients suitable for both dermal and ophthalmic applications and had previously been shown to increase oral, dermal or ocular bioavailability of curcumin, respectively [26,27]. However, a study comparing the bioavailability of curcumin from these formulations directly to each other was not available and was therefore tested in this study.

The bioavailability of the formulations was first tested on the already established ex vivo skin model to distinguish their penetration efficacy. In the next step, the formulations showing significant differences in the penetration of curcumin in the skin model were selected for corneal penetration studies. For this, instead of using skin, the selected formulations were applied on whole porcine eyeballs [28]. After incubation for 30 min, the corneal tissues were separated and treated similarly to the pig ears and cross-sectional images were taken on an inverted epifluorescence microscope. The images obtained were subsequently subjected to digital image analysis as carried out with the images obtained from the dermal penetration testing (Figure 1). Finally, the results obtained from both methods were compared to each other.

## 2. Materials and Methods

### 2.1. Materials

Curcumin, as *Curcuma longa* L. dry extract (80% curcumin; 95% curcuminoids), was obtained from Receptura Apotheke (Cornelius-Apothekenbetriebs-OHG, Frankfurt am Main, Germany). The oil used was Miglyol^®^ 812 (medium chain triglycerides (MCT) from Caesar & Loretz GmbH (Hilden, Germany). As stabilizers and surfactants, Polysorbate 80 (Tween^®^ 80, VWR International GmbH, Darmstadt, Germany), water-soluble vitamin E (d-α-tocopherol polyethylene glycol 1000 succinate (TPGS) from Gustav Parmentier GmbH (Frankfurt am Main, Germany)) and egg lecithin (Lipoid^®^ E80, phospholipid from egg yolk with 80% phosphatidyl choline) from Lipoid GmbH (Ludwigshafen, Germany) were used. Beta-cyclodextrin was obtained from BLD Pharmatech Ltd. (Shanghai, China) and absolute ethanol (HPLC grade ≥ 99.8%) was from Sigma-Aldrich Chemie GmbH (Steinheim, Germany). Purified water was freshly obtained from a PURELAB^®^ Flex 2 (ELGA LabWater, Veolia Water Technologies Deutschland GmbH, Celle, Germany) and a Millipore Milli-Q lab water system (Merck, Darmstadt, Germany).

### 2.2. Methods

#### 2.2.1. Preparation of the Formulations

The formulations used in this study included an oily solution of curcumin, an oily suspension of curcumin, curcumin nanocrystals (aqueous curcumin nanosuspension), curcumin micelles, curcumin liposomes and curcumin loaded into cyclodextrins. All formulations were prepared according to previously established protocols [26,27,29]. The composition of the formulations is shown in Table 1 and the preparation of each formulation is briefly described below.

The oily solution and suspension were prepared by adding the curcumin bulk powder to the oil while stirring. Nanocrystals were prepared using small-scale bead milling. The bead milling was carried out for 14 h in a 25 mL Erlenmeyer flask placed on an ice bath and equipped with 1.0–1.2 mm yttria-stabilized zirconia grinding beads (SiLibeads^®^, Sigmund Lindner GmbH, Warmensteinach, Germany) and a magnetic stirring bar (Asteroid^®^ 25, 2mag AG, München, Germany). The bead/suspension ratio was 40/60 (*v*/*v*) and the stirring speed was 1500 rpm. Micelles were produced by preparing an aqueous Polysorbate 80 solution to which the curcumin bulk powder was added while stirring. Liposomes were produced by adding the curcumin powder being dissolved in ethanol (0.5 g curcumin in 4.5 g ethanol) to empty pre-prepared liposomes with subsequent high-pressure homogenization at 500 bar (3 cycles, room temperature, LAB 40, APV, Unna, Germany). Empty liposomes were pre-prepared by adding phospholipids to water that was heated to 40 °C. The mixture obtained was high-speed stirred with an Ultra Turrax (IKA, Staufen, Germany) for 30 s at 25,000 rpm and then high-pressure homogenized at 500 bar (3 cycles, with an LAB 40, APV, Unna, Germany). Cyclodextrins were loaded with curcumin by mixing curcumin bulk powder and cyclodextrin at a ratio of 1:1 (*w*/*w*). The mixture was dissolved in ethanol to obtain a clear solution. The preparation was then allowed to evaporate overnight at room temperature. The cyclodextrin complexes obtained were then pulverized and sieved with a # 80 sieve. Prior to application, the curcumin-loaded cyclodextrin powder was diluted with water to obtain a cyclodextrin formulation containing 1% (*w*/*w*) curcumin [26,27].

#### 2.2.2. Characterization of the Formulations

All formulations were subjected to light microscopy using an Olympus BX53 light microscope (Olympus Cooperation, Tokyo, Japan), which was equipped with an Olympus SC50 CMOS color camera (Olympus Soft Imaging Solutions GmbH, Münster, Germany). The particle size of all particulate formulations was measured using laser diffractometry (LD) and dynamic light scattering (DLS). The particle size of the oily suspension was assessed in the original dispersion medium (MCT). LD analysis was carried out using a Mastersizer 3000 (Malvern Panalytical Ltd., Malvern, UK). Mie theory was used for data analysis and the real refractive index was set to 1.87. The imaginary refractive indices used were 0.1 for the blue light (470 nm) and 0.01 for the red light (632.8 nm) laser beam. DLS analysis was performed with a Zetasizer Nano ZS (Malvern Panalytical Ltd., Malvern, UK), and data analysis was carried out in general purpose mode.

#### 2.2.3. Dermal Penetration Studies

Dermal penetration efficacy was determined using a previously established ex vivo porcine ear model with subsequent digital image analysis [11]. The procedure was as follows: Fresh pig ears, obtained from a local slaughterhouse, were washed with lukewarm water (approx. 23–25 °C) and gently dried with a paper towel. The ears were examined and scratch-free skin areas with a diameter of 1.5 cm on the dorsal side of the ears were identified and marked with a stamp. Any hair in these areas was carefully trimmed to a length of about 1–3 mm. A volume of 13.25 µL of each formulation (Table 1) with and without curcumin (i.e., only the vehicle) was applied to the skin areas without massage. The amount applied corresponded to 7.5 µL/cm^2^ and thus represented a finite dose setup, i.e., a dose < 10 µL/cm^2^, which is used to mimic real-world applications [30].

After application, the ears were incubated for 2 h at 32 °C, before removing them from the oven and carefully rinsing off the formulations. Ears were carefully dabbed dry with a paper towel, and punch biopsies (12 mm diameter) were taken from the treated skin areas. For comparison, punch biopsies were also taken from untreated skin before (blank _initial_) and after incubation (blank _after_), respectively. The freshly obtained skin biopsies were immediately embedded in Tissue-Tek^®^ O.C.T.™ (Sakura Finetek Europe B.V., Alphen aan den Rijn, The Netherlands), frozen at −40 °C and stored at −18 °C until further use.

In the next step, the frozen skin biopsies were cut into 20 µm thick vertical skin sections with a cryomicrotome (Frigocut 2700, Reichert-Jung, Nußloch, Germany), placed on slides and analyzed with an inverted epifluorescence microscope (Olympus CKX53 equipped with an Olympus DP22 color camera, Olympus Deutschland GmbH, Hamburg, Germany). The selected fluorescence filter for the analysis was the DAPI HC filter block system (excitation filter: 460–500 nm, dichroic mirror: 500 nm, emission filter: from 500 nm (LP)). The intensity of the fluorescent light source was set to 50% and the exposure time was adjusted to 50 ms. The settings were kept constant during analysis and the experiments were carried out in triplicate.

#### 2.2.4. Corneal Penetration Studies

Corneal distribution of curcumin was evaluated ex vivo using a previously established ex vivo porcine whole-eye model [7]. For this, porcine eyes were collected from the abattoir (DaHua Supermarket, Auckland, New Zealand) and transported to the laboratory in cold PBS. All eyes were inspected for corneal damage or lacerations and only healthy eyes were used for penetration studies after trimming off any excess tissue surrounding the eyes. The eye model was set up by placing the eyes into 6-well microplates lined with a non-absorbent lint-free sheet (DAY-LEE™, Johnson & Johnson Medical, New Brunswick, NJ, USA) soaked in Hank’s Balanced Salt Solution (HBSS; ThermoFisher Scientific Auckland, New Zealand). Custom-made polycarbonate corneal sleeves with an internal diameter of 18.5 mm (manufactured by the University of Auckland Biomedical Engineering Workshop, Auckland, New Zealand) were placed on top of each eye, such that the cornea and bulbar conjunctiva were exposed. Excised eyelid tissue was placed on top of each eye with the tarsal conjunctiva facing the cornea. Eyes were incubated at 35 ± 3 °C and, after equilibration for 15 min, 300 µL of the test formulation was applied between the tarsal conjunctiva and cornea. After 15 min, eyes were rinsed and washed with HBSS to remove any excess formulation. Afterward, the cornea of each eye was carefully excised with scissors and a scalpel and fixed in Tissue-Tek^®^ O.C.T. compound (Sakura^®^ Finetek, Torrance, CA, USA). The tissues were sectioned (CryoStar NX50, Thermo Scientific, Waltham, MA, USA) to a thickness of 10 µm and observed under a fluorescent microscope (Apotome.2, Zeiss, Jena, Germany). For each corneal sample, 12 sections from the central regions were imaged at 5 positions each, resulting in a total of 60 images for each cornea. A 475 nm LED laser, with laser intensity at 25%, was used to illuminate the tissue sections, and the signal was collected using the camera objective (20 × 0.5 Axiocam 506, Zeiss, Jena, Germany). The built-in green fluorescence filter (FITC-109; BP 450–490/BS495/BP 500-500) with an exposure time of 1000 ms and 100% intensity was used. The settings were kept constant during analysis and the experiments were carried out in triplicate.

#### 2.2.5. Digital Image Analysis

The microscopic images obtained were subjected to digital image analysis to determine the penetration efficacy of curcumin from the different formulations into the skin and the cornea. The analysis was carried out via ImageJ software version 1.8.0 [31,32]. In the first step, images were subjected to an automated RGB threshold procedure, which was used to eliminate the autofluorescence of the skin [11]. The remaining pixels after the threshold algorithm represent the penetrated curcumin into the skin. The amount of penetrated curcumin (TAP_Cur_) was determined from these images semi-quantitatively by analyzing the mean grey value per pixel (MGV/px) in each image. The higher the MGV/px, the more curcumin penetrated into the skin. In addition, the mean penetration depth (MPD) was determined. This was carried out by manually measuring the distance between the skin/cornea surface and the most distant pixels from the surface. Finally, for the skin samples, the stratum corneum thickness (SCT) was measured from (i) untreated skin sections (blank _initial_ and blank _after_), (ii) skin sections treated with vehicles only and (iii) skin sections treated with the curcumin formulations. The SCT was determined from the original images prior to the RGB threshold procedure. The procedure was similar to the measurement of the MPD, i.e., the scale function of ImageJ software was used to manually measure the SCT in each image.

#### 2.2.6. Statistical Analysis

JASP software (version 0.13.1) was used for discriminative statistics and comparison of the mean values [33]. Tests for normal distribution and variance homogeneity were performed with the Shapiro–Wilk and Levene’s tests, respectively. The means of normally distributed data were then compared with an ANOVA, which was Welch adopted in case of variance heterogeneity. Dunnett and Games–Howell post hoc tests were also conducted. Non-parametric data were subjected to a Kruskal–Wallis analysis of variance, followed by Dunn’s post hoc tests.

In some cases, a direct comparison of two data sets was deemed important. In this case, the Student’s *t*-test for independent samples was used for normally distributed data and the Mann–Whitney test was performed for non-normally distributed data. A *p*-value < 0.05 was defined as statistically significant. Error bars in the figures represent the standard deviation.

## 3. Results

### 3.1. Formulation Preparation and Characterization

**Figure 2:**
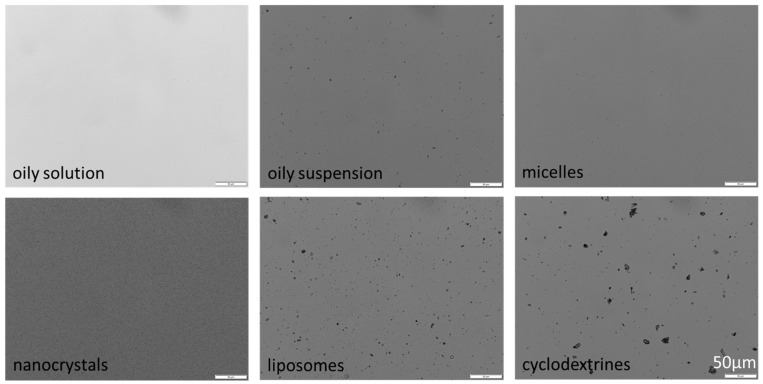
Microscopic images of curcumin formulations.

**Figure 3:**
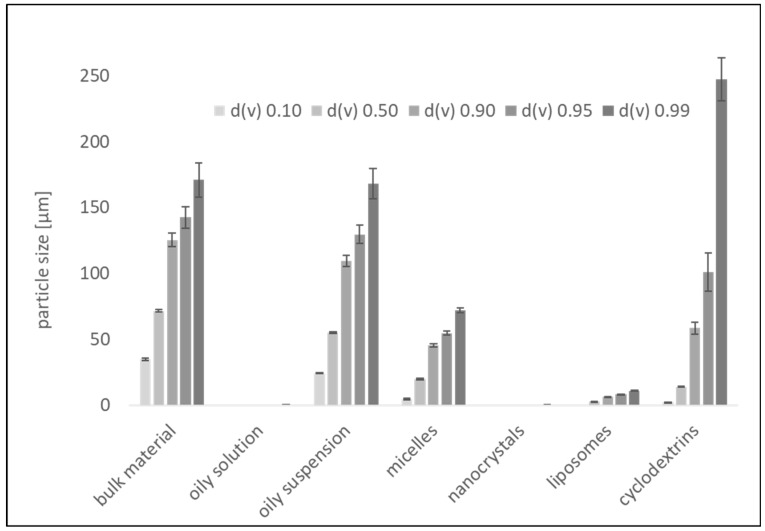
Particle size analysis (LD data) of curcumin formulations.

While the oily solution, containing only 0.1% curcumin, appeared clear (Figure 2), LD analysis revealed a very small amount of larger undissolved particles corresponding to the size of the bulk material (Figure 3). The oily suspension (1.0% curcumin) contained particles that were slightly smaller than the bulk material, indicating that all particles had started to dissolve in the oil until the saturation solubility of curcumin was reached. The d(v)0.90, d(v)0.95 and d(v)0.99 were similar to the bulk material and to the few remaining particles in the oily solution.

The nanocrystals possessed a particle size of 172 ± 3 nm (DLS data) and LD measurements and light microscopy confirmed the absence of particles >1 µm (Figure 2 and Figure 3). Micelles could solubilize almost all of the curcumin. However, similar to the oily solution, a few larger particles remained. The particle size of these remaining particles was smaller than that of the particles from the bulk material and also much smaller than the size of the remaining particles in the oily suspension, suggesting increased solubility of curcumin due to the presence of micelles, which allows the large curcumin particles to dissolve more thoroughly, albeit not completely.

Due to the remaining larger particles in the micelle formulation, DLS analysis detected the small-sized micelles and the larger particles, which led to a combined size with a z-average of about 700 nm and a polydispersity index (PDI) > 0.8. The liposomal formulation was found to be unstable. Already shortly after preparation, larger particles became clearly visible within the formulation. DLS analysis revealed a particle size of about 1360 nm with a PDI being >0.7. The large size and broad particle size distribution were also confirmed by LD analysis and light microscopy (Figure 2 and Figure 3). The cyclodextrin formulation contained the largest particles of all formulations as well as the highest number of larger-sized curcumin particles (Figure 2 and Figure 3), due to the unentrapped curcumin, which was dissolved in ethanol during preparation, but precipitated into large crystals during the drying step.

Overall, only the oily suspension and the curcumin nanocrystals were formulated optimally. All other formulations, due to the presence of larger curcumin particles, were considered to be not formulated optimally. Nonetheless, due to the different solubilization approaches used, differences in curcumin penetration efficacy were expected, which was tested in the next part of the study.

#### Dermal Penetration Efficacy

**Figure 4:**
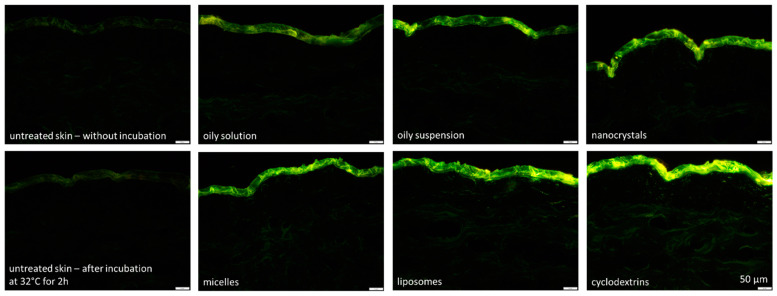
Fluorescence images of tissue cross-sections showing untreated skin and skin treated with the different curcumin formulations. Scale bar = 50 µm.

**Figure 5:**
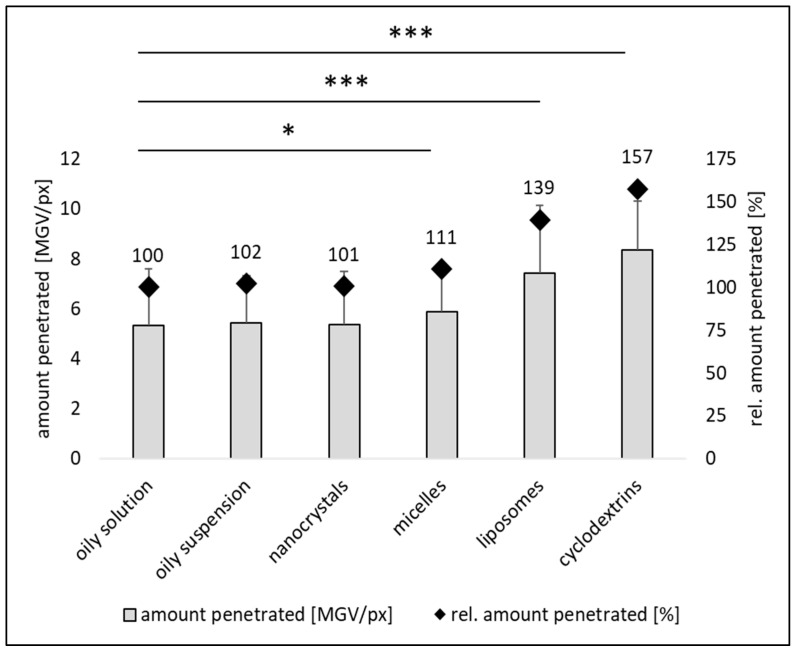
Dermal penetration efficacy [MGV/px] and relative (rel.) penetration efficacy [%] of curcumin from different formulations. *: *p*-value < 0.05; ***: *p*-value < 0.001.

**Figure 6:**
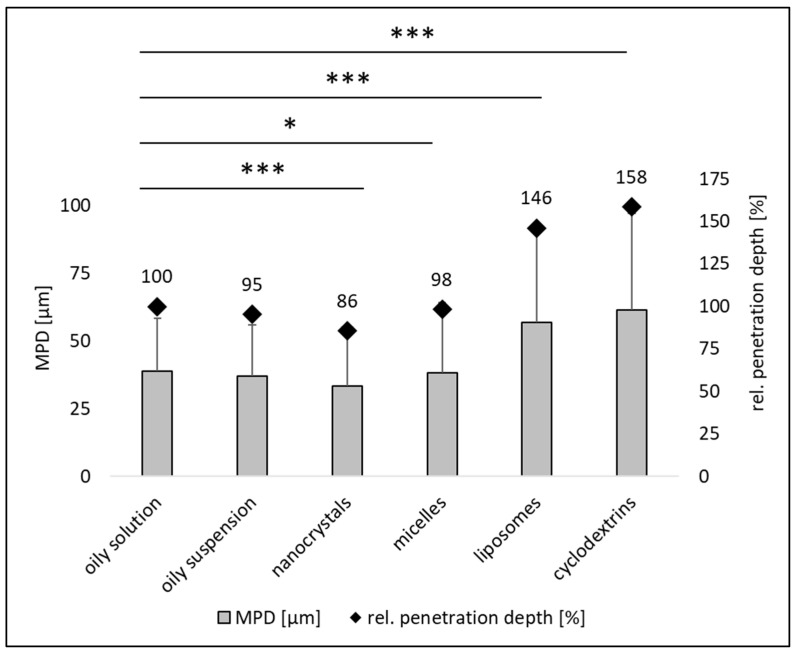
Mean dermal penetration depth [µm] and relative (rel.) penetration depth [%] of curcumin from different formulations. *: *p*-value < 0.05; ***: *p*-value < 0.001.

Cross-sectional images of skin biopsies clearly showed that the various formulations resulted in different dermal penetration efficacies of curcumin (Figure 4), which was subsequently confirmed by digital image analysis (Figure 5 and Figure 6).

The amount of dermally penetrated curcumin was similar for the oily solution, the oily suspension and the nanocrystals (Figure 5). The micelles and nanocrystals led to slightly higher penetration; however, the difference was not statistically significant. The liposomes and the cyclodextrin formulation resulted in significantly higher curcumin penetration when compared to the other formulations with the cyclodextrin formulation being the most effective (Figure 4, Figure 5 and Figure 6). In future, the penetration efficacy of the cyclodextrin formulation could, according to Yadav et al., be further enhanced by an increase in ß-cyclodextrin over the chosen 1:1 ratio and or by using a chemically modified ß-cyclodextrin and a more sophisticated production technique [29].

The analysis of the penetration depths showed no differences in penetration depth between the oily solution, the oily suspension and the micelles, but found that the nanocrystals led to a slightly lower penetration depth when compared to the oily solution (Figure 6). The MPD for liposomes and the cyclodextrin formulation was deeper with curcumin incorporated into the cyclodextrins penetrating the deepest (Figure 6) [34].

In the next step, the penetration depth of the formulations was compared to the SCT (Table 2) to determine the skin layer into which curcumin was delivered, i.e., an MPD > SCT means that curcumin penetrated across the stratum corneum, whereas an MPD < SCT means that curcumin remained in the stratum corneum. Results showed that all formulations were able to transport curcumin across the stratum corneum, i.e., the MPD was larger than the SCT for all formulations.

The next step evaluated if curcumin was able to penetrate into the viable dermis, which would allow curcumin to enter the systemic circulation. The epidermis in the ex vivo model has a thickness of about 100 µm. Hence, an MPD > 100 µm would indicate that curcumin penetrated the viable dermis. However, none of the formulations resulted in an MPD > 62 µm, suggesting that none of the formulations was able to transport curcumin into the viable dermis.

### 3.2. Influence on Stratum Corneum Thickness

**Figure 7:**
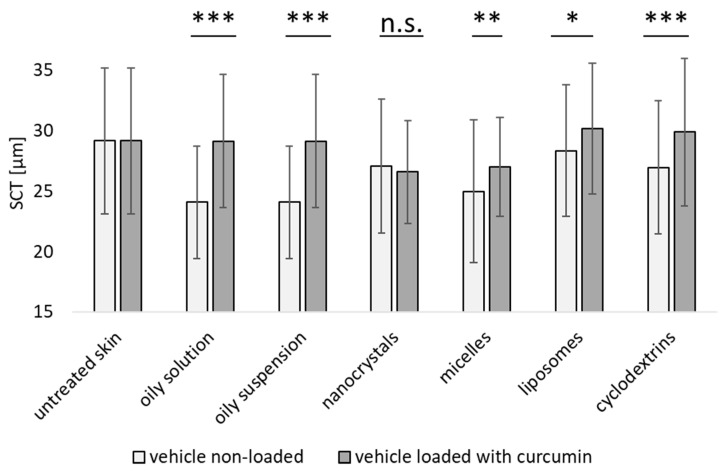
Influence of unloaded vehicles and curcumin-loaded formulations on SCT. *: *p*-value < 0.05; **: *p*-value < 0.01, ***: *p*-value < 0.001, n.s.: non-significant.

The results from the dermal penetration analysis were unexpected. Especially for the oily suspension and the nanocrystals, a much higher penetration of curcumin was expected in comparison to the oily solution [35]. Hence, more information was needed to be able to better understand the results obtained from the dermal penetration testing. Therefore, in the next step, the SCT was measured for untreated skin and for the skin treated with the various vehicles without curcumin. The results from those skin cuts were then compared to the SCT of skin treated with curcumin-containing formulations (Figure 7 and Table 3).

The SCT is a sensitive measure of possible changes in the stratum corneum, e.g., it allows us to detect irritating, dehydrating or hydrating effects caused by the skin treatment. Dehydration means that the SCT is thinner than that of untreated skin and consequently, a thicker SCT, when compared to untreated skin, means that the skin is more hydrated than untreated skin [11].

Results indicated that treatments with vehicles without curcumin had a slightly dehydrating effect on the skin, i.e., the SCT after treatment with the unloaded vehicles was thinner than that of untreated skin (Figure 7 and Table 3A).

The effect was most pronounced for the oily solution and suspension, indicating that the low amount of oil was unable to cause an occlusive effect on the skin. This was due to the finite dose setup, meaning that the amount of oil was too small to cover the skin with an occlusive oil layer that was thick enough to prevent water evaporation from the skin. The dehydrating effects of the micelles (composed of Polysorbate 80), the surfactant used for the stabilization of the nanocrystals (TPGS) and the cyclodextrins can be explained by their hygroscopic properties. Both surfactants contain hygroscopic polyethylene glycol units and cyclodextrins are hygroscopic due to their sugar moieties [36,37,38].

As hygroscopic excipients are unable to fully penetrate the skin, it is highly likely that parts of them remain unabsorbed on top of the skin, thus pulling out water from the stratum corneum. This may have ultimately led to dehydration of the skin and, consequently, a thinner SCT when compared to skin that was not treated with these excipients (Table 3A). Treatment with the empty liposomes did not cause a dehydrating effect on the skin.

The addition of curcumin increased the SCT, when compared to the unloaded vehicles for all formulations, except for the curcumin nanocrystals (Figure 7 and Table 3B), for which the thickness remained similar to untreated skin (Table 3C). The increase in SCT after the addition of curcumin, except for the curcumin nanocrystals, can be explained by the hygroscopic properties of curcumin [22]. When considering the hygroscopic effects of curcumin, it can be assumed that curcumin that penetrates the skin will unfold its hygroscopic properties within the skin. Hence, the SCT will increase as the amount of curcumin in the skin increases, attracting more water into the stratum corneum. In contrast, curcumin remaining on top of the skin will remove water from the skin (and/or the formulation), which then causes a reduced SCT, as seen for the oily solution and suspension.

The either hydrating or dehydrating effect of curcumin on the stratum corneum can be best seen when comparing the results obtained from the oily curcumin solution (0.1%) and suspension (1.0%). Although the amount of curcumin penetrated was similar for both formulations (Figure 5 and Figure 6), one would have expected that the suspension would lead to higher drug penetration. However, the penetration depth was slightly less for curcumin in suspension than for the solution (*p*-value > 0.05) and the SCT of the skin treated with the suspension was much thinner than that of the oily solution (*p*-value < 0.001) [35,39].

The results indicate that curcumin penetration from these formulations occurred via the solvent drag mechanism. Hence, the oil penetrated the skin and the curcumin being dissolved in the oil was soaked (dragged) into the skin with the solvent [40]. The amount of oil penetrating the skin and the amount of curcumin being dissolved in the oil can be assumed to be very similar for both formulations. However, the amount of curcumin remaining on top of the skin was much higher for the curcumin suspension, with the remaining hygroscopic curcumin removing water from the stratum corneum causing a thinner SCT than the oily solution, which kept the water inside the skin [41]. In addition, the water pull-out effect of curcumin may also create a push-out effect for the API, which thus hampers effective penetration of curcumin from the outside to the inside of the skin. The trend was also apparent when comparing the higher MPD of the oily solution with the lower MPD of the oily suspension (Figure 6 and Table 2).

The SCT of the nanocrystal-treated skin was the thinnest because this formulation contained curcumin particles that could not penetrate the skin [35]. The small size of the nanocrystals, in comparison to the large size of the particles in the oily solution, creates a much larger surface and also the number of particles is much higher for the nanocrystals than for the other formulations. Therefore, it is highly reasonable that the nanocrystals created a much stronger hygroscopic effect than the oily suspension and the other formulations. Hence, this formulation was the one with the most pronounced pull-out effect, thus, leading to the thinnest SCT.

The penetration of curcumin from the micelles was slightly better than the penetration from the oily formulations, but the SCT was thinner, hence, the formulation had a more dehydrating effect on the skin. A possible explanation is that the micelles contained a 10-fold higher amount of curcumin when compared to the oily solution as well as hygroscopic Polysorbate 80. It can be considered that most of the hygroscopic curcumin and the hygroscopic Polysorbate 80 remained on the skin surface, thus causing a stronger pull-out effect for water, which then caused the reduced SCT when compared to the oily solution and suspension (Figure 6 and Table 3). The liposomes and the cyclodextrins allowed for much better penetration of curcumin into the skin, but at the same time, due to the much higher amount of curcumin in the formulations, also larger amounts of curcumin were left outside the skin. The curcumin in the skin caused the stratum corneum to swell, whereas the curcumin remaining on top of the skin likely caused a pull-out effect. Both effects seem to be balanced, i.e., they seem to cancel each other out, so the SCT of both formulations was similar to the one of untreated skin.

### 3.3. Corneal Penetration Efficacy of Formulations

The comparison of the dermal penetration efficacies of curcumin from the different formulations showed that oily solution, oily suspension and nanocrystals led to similar penetration efficacies (Figure 5 and Figure 6). The micelles showed slightly better penetration than the oily solution, while liposomes and cyclodextrins showed significantly greater penetration. Hence, based on their penetration efficiency, the six formulations were combined into three groups, each having significantly different dermal penetration. Group A showed reasonably good penetration of curcumin (oily solution, oily suspension and nanocrystals). Group B showed relatively greater penetration of curcumin (micelles) than Group A and Group C, which showed enhanced curcumin penetration (liposomes and cyclodextrins). Based on these results, the next step was to select appropriate formulations from each group that would be suitable for testing the corneal penetration efficacy. The aim of this step was to investigate if the digital image processing method used for the skin could also be applied to analyze the corneal penetration efficacy of active compounds using an ex vivo porcine eye model to discriminate between “good” and “bad” performing formulations. Based on the results from the dermal penetration testing, one formulation of each group that led to significant differences in the penetration of curcumin in the skin model was selected for the testing of the corneal penetration efficacy. Thus, the oily solution, micelles and cyclodextrin formulation were selected from Groups A, B and C, respectively. Liposomes were not selected for further testing due to their physical instability.

**Figure 8:**
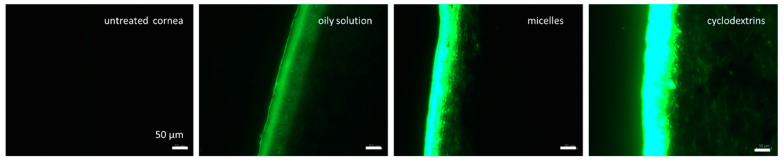
Fluorescence images of untreated cornea and cornea treated with different curcumin-containing formulations. Scale bar = 50 µm.

**Figure 9:**
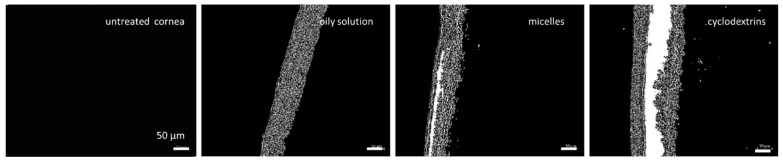
Fluorescence images after RGB threshold of untreated cornea and cornea treated with different curcumin-containing formulations. Scale bar = 50 µm.

For this study, an ex vivo porcine eye setup was used [28,42], and, post-incubation with the test formulation, the corneal tissue was processed in the same manner as the dermal tissues. The corneal sections were imaged using inverted epifluorescence microscopy (Figure 8). Thus, the adopted procedure from the skin model could be adequately applied to corneal tissues and the tissue processing method could be successfully transferred from the ex vivo skin model to the ex vivo eye model. In the next step, the images obtained were subjected to the automated RGB threshold procedure (Figure 9).

**Figure 10:**
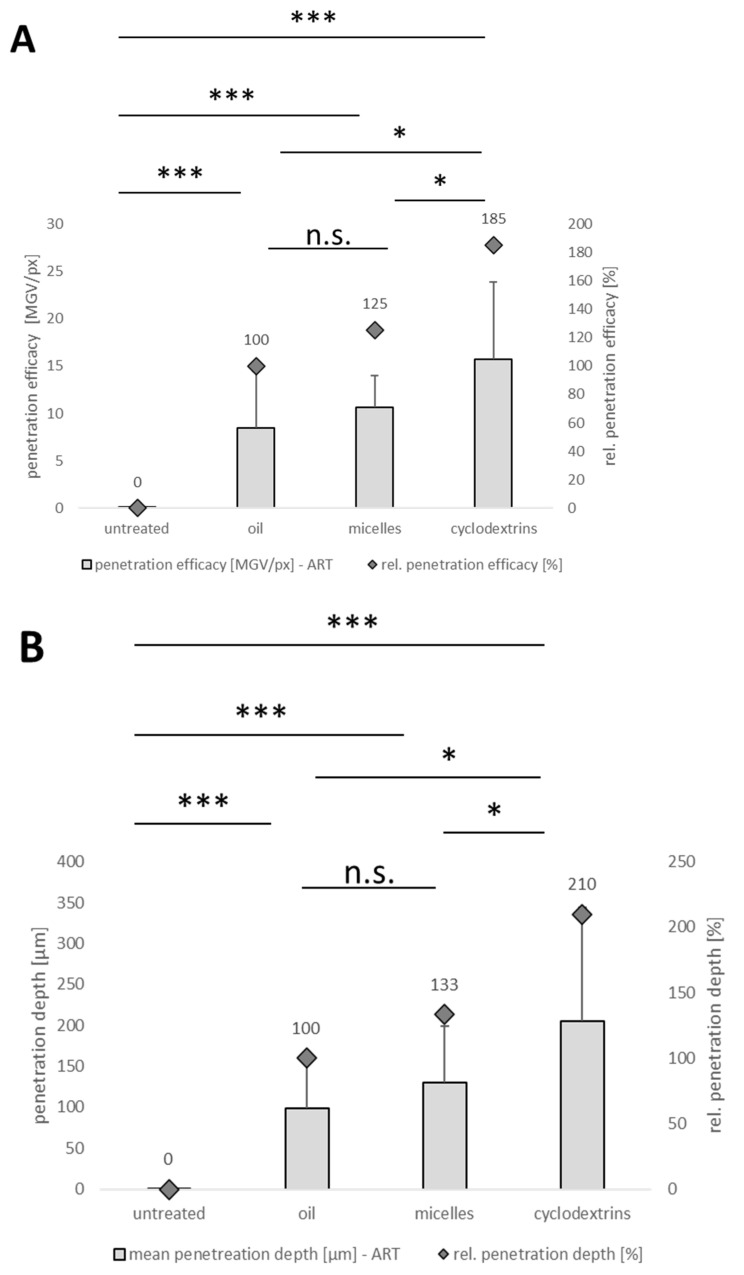
Influence of formulation principle on corneal penetration efficacy of curcumin—data analysis from images obtained after automated RGB threshold. (**A**) Corneal penetration efficacy [MGV/px] and rel. penetration efficacy [%] of curcumin from different formulations. (**B**) Mean penetration depth [µm] and rel. penetration depth [%] of curcumin from different formulations. *: *p*-value < 0.05, ***: *p*-value < 0.001, n.s.: non-significant.

From the images obtained, the amount of curcumin penetrating the cornea and the MPD were determined using ImageJ software (Figure 10). After the digital RGB threshold, which subtracts the autofluorescence of the tissue from the image, the remaining white pixels in the picture correspond to the penetrated active compound [11]. Consequently, for non-treated corneas, no pixels remained after the digital RGB threshold, while all other images contained significantly higher amounts of white pixels (Figure 9 and Figure 10).

In line with our observations in the skin, the oily solution was the least efficient formulation, with the lowest MGV/px value. Micelles showed relatively higher penetration, while the cyclodextrin formulation showed the greatest corneal penetration. These results are also in agreement with previous ocular penetration studies performed with such formulations [9,43,44]. Our data demonstrate that the digital image processing method, which was initially developed for the testing of dermal penetration efficacy, can also be applied to evaluate the penetration of active compounds into other tissues, such as the cornea, as shown in this study.

When comparing the images of untreated skin to the images of untreated cornea (Figure 4 and Figure 8), it can be seen that the cornea, in comparison to the skin, has relatively lower autofluorescence. This can be explained by the fact that the cornea contains relatively fewer layers of cells within the corneal stroma which predominantly comprises of water, and makes up the majority of the cornea [45]. As such, the porcine corneal tissue reportedly has a hydration level of approximately 78–82% [7,46]. Since water has no autofluorescence, the high water content of the cornea reduces the autofluorescence of this tissue when compared to the skin.

**Figure 11:**
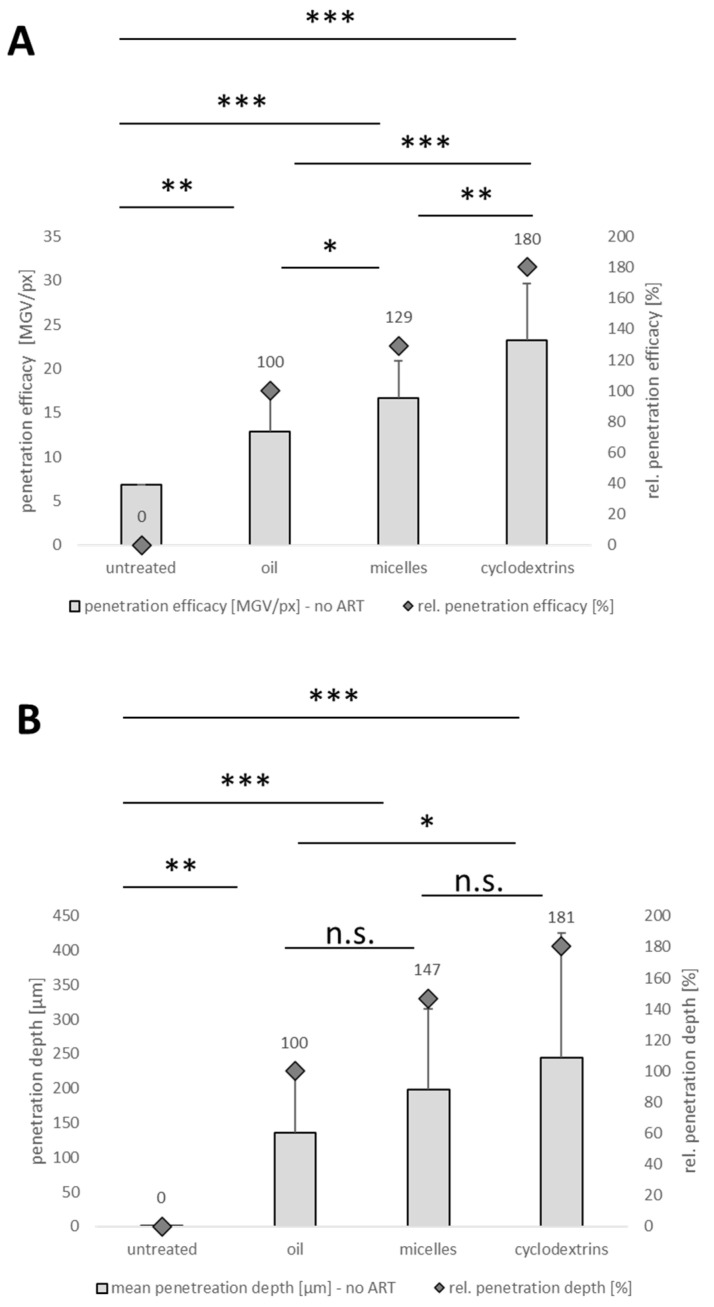
Influence of formulation principle on corneal penetration efficacy of curcumin—data analysis from original images obtained from inverted epifluorescence microscopy. (**A**) Corneal penetration efficacy [MGV/px] and rel. penetration efficacy [%] of curcumin from different formulations. (**B**) Mean penetration depth [µm] and rel. penetration depth [%] of curcumin from different formulations. *: *p*-value < 0.05; **: *p*-value < 0.01, ***: *p*-value < 0.001, n.s.: non-significant.

Due to the low autofluorescence of the untreated cornea (Figure 8), it was hypothesized, that digital RGB thresholding is not needed for corneal tissue. This renders analysis of corneal penetration efficacy even more convenient than analysis of dermal penetration efficacy because there would be no need to program the automated RGB threshold macro, which requires special training and some programming expertise with the ImageJ software [11]. Hence, the analysis could be performed directly on the original images obtained via inverted epifluorescence microscopy. To confirm this hypothesis, in the next step, the penetration efficacy and the penetration depth of curcumin from the different formulations were assessed from the original images that were not subjected to the automated RGB thresholding (Figure 11).

Results of the image analysis without RGB thresholding were comparable to those obtained from the image analysis after RGB thresholding, i.e., all cornea tissues that were treated with curcumin formulations were found to have a significantly higher number of pixels in the images. Within the curcumin-treated corneas, similar to the analysis of the images after RGB thresholding, the highest amount of curcumin penetrated from the cyclodextrin formulation and the least amount from the oily solution (Figure 11A). Hence, our data indeed suggest that the penetration analysis into the cornea can be performed without the RGB thresholding step.

Interestingly, in contrast to the analysis with the RGB thresholding procedure, analysis with the original images resulted in a significant penetration efficacy result between the oily solution and the micelles, whereas the *p*-value was >0.05 when the images were analyzed with the RGB threshold procedure (Figure 10A). This indicates that the analysis without RGB threshold analysis is even more discriminative than the method with RGB threshold procedure. This can be explained by the fact that the RGB threshold procedure eliminates tissue autofluorescence by eliminating pixels from the images that do not exceed a specific intensity. Consequently, it can also eliminate some low-intensity pixels that correspond to the penetrated active compound, leading to reduced sensitivity of the analysis [11]. The RGB threshold procedure is mandatory if tissue with high autofluorescence is used for the analysis, which is the case for skin when testing dermal penetration [11]. However, when testing tissue with low autofluorescence, the analysis without the RGB threshold procedure was found to be more discriminative and also more convenient.

In contrast to the above results, the RGB threshold procedure led to more discriminative MPD analysis results than image analysis without RGB thresholding (Figure 10B and Figure 11B). The reason for this is that the low autofluorescence of the cornea, although very weak, blurs the images. Moreover, the corneal epithelium and stroma differ significantly in terms of their hydrophobic–lipophilic balance and water content, which can lead to considerable discrepancies when comparing drug penetration depth [47,48]. This means that clear discrimination between pixels that correspond to the penetrated curcumin and those that correspond to corneal autofluorescence is difficult to perform. Discrimination between black and white pixels is relatively easy because the differences are clearly visible, making MPD determination from images with RGB thresholding more discriminative. It is worth noting that, unlike the skin samples, blank corneal tissues used for generating a threshold RGB value and corneal tissue samples used for penetration studies are not obtained from the same animal; thus, background corneal autofluorescence between different corneal tissue samples may vary considerably.

Overall, our results suggest that corneal penetration efficacy can be determined from the original images without the application of the RGB threshold procedure, which is mandatorily needed for testing dermal penetration efficacy. The assessment of the corneal penetration efficacy without the RGB threshold procedure is faster and simpler and leads to even more discriminative results in corneal tissues. However, when the penetration depth is being analyzed, the images after the RGB threshold should be used to compensate for the difference in autofluorescence in different regions of the cornea.

As a final step in dermal penetration analysis, the SCT was determined, which can provide meaningful additional information and help to understand the underlying mechanisms of API penetration into the skin in more detail. However, the corneal tissue is extremely resistant to change in thickness and exchange of water, since even minor fluctuations in water content can have a significant effect on corneal transparency and visual acuity [47]. Significant changes in water content typically correlate with loss of epithelial and/or endothelial barrier properties [47,49]. Moreover, thickness measurements in the cornea are difficult due to poor autofluorescence of the corneal tissue. Thus, comparative mechanistic studies that investigate the underlying mechanisms of drug penetration in the skin and cornea could not be performed. However, despite significant differences in the mechanism of drug penetration into the skin and the corneal tissues, our digital imaging and processing method could be applied to test the penetration efficacy of fluorescent APIs, such as curcumin, from different formulations. Curcumin, a BCS class II drug, was used as the model hydrophobic drug in this study. Further studies with different compounds having different hydrophilic–lipophilic balances may be useful in further demonstrating the versatility of this method.

## 4. Conclusions

Curcumin is a poorly soluble compound that needs appropriate formulations to sufficiently improve the solubility and bioavailability of this compound. In this study, curcumin was formulated as an oily solution, oily suspension and aqueous nanosuspension. In addition, it was encapsulated into micelles, liposomes and cyclodextrins. The dermal penetration efficacy of curcumin from all six formulations was assessed. Three groups, each representing a different penetration efficacy and mechanism for curcumin, were identified. The least penetration was found for the oily solution, oily suspension and aqueous nanosuspension. A slightly higher penetration was found for the micelles and the most efficient dermal penetration was found for the liposomes and the cyclodextrins.

Corneal penetration was tested by selecting one formulation from each group using the ex vivo porcine eye model with subsequent digital image analysis to semi-quantitatively evaluate corneal penetration. Results obtained from testing the different curcumin formulations on the cornea were in line with those found for dermal penetration efficacy. Hence, the results could confirm that the digital image analysis technique can indeed be repurposed for ocular applications. Interestingly, it was found that the application of the digital image analysis for the testing of corneal penetration was even simpler because the automated RGB threshold procedure was not needed due to the low autofluorescence of the cornea.

The results of the study demonstrate that digital image processing of the ex vivo skin model and the ex vivo eye model can serve as swift and economical tools for evaluating the potential of dermal and ophthalmic formulations, respectively. This technique can be effectively used to evaluate the penetration and distribution of fluorescent active compounds into the skin and/or into the corneal tissue. The penetration models and quantification techniques detailed in this study provide a promising cost-effective and versatile tool for further studies and have the potential to advance future development in this field. For curcumin, cyclodextrins were identified as the most effective formulation principle for both dermal and corneal penetration.

## Figures and Tables

**Table 1:** Overview of formulations and their compositions tested.

Formulation	Amount of Curcumin [% *w*/*w*]	Other Excipients [% *w*/*w*]
oily solution	0.1	MCT 99.9
oily suspension	1.0	MCT 99.0
nanocrystals	1.0	TPGS 1.0, water 98.0
micelles	1.0	Tween 80 1.0, water 98.0
liposomes	1.0	phospholipid 1.0, water 98.0
cyclodextrins	1.0	ß-cyclodextrins 1.0, water 98.0

**Table 2:** Influence of formulation principles on MPD and SCT (mean ± SD).

Formulation	MPD [µm] ± SD	SCT [µm] ± SD	Δ MPD-SCT [µm]
oily solution	39 ± 2	29 ± 6	−10
oily suspension	37 ± 2	29 ± 6	−8
nanocrystals	33 ± 2	27 ± 4	−7
micelles	38 ± 2	27 ± 4	−11
liposomes	57 ± 3	30 ± 5	−27
cyclodextrins	62 ± 2	30 ± 6	−32

**Table 3:** Influence of unloaded vehicles and curcumin-loaded formulations on relative SCT. *: *p*-value < 0.05; **: *p*-value < 0.01, ***: *p*-value < 0.001, n.s.: non-significant.

	(A)		(B)		(C)	
	SCT of Unloaded Vehicle to SCT of Untreated Skin		SCT of Formulation to SCT of Unloaded Vehicle		SCT of Formulation to SCT of Untreated Skin	
untreated skin	100		100		100	
oily solution	83	***	133	***	109	**
oily suspension	83	***	121	***	100	n.s.
nanocrystals	93	*	98	n.s.	91	***
micelles	86	***	108	**	93	**
liposomes	97	n.s.	106	*	103	n.s.
cyclodextrin	92	**	111	***	103	n.s.

## Data Availability

The original contributions presented in this study are included in the article, and further inquiries can be directed to the corresponding authors.
